# Human lower leg muscles grow asynchronously

**DOI:** 10.1111/joa.13967

**Published:** 2023-11-02

**Authors:** Brian V. Y. Chow, Catherine Morgan, Caroline Rae, David I. Warton, Iona Novak, Suzanne Davies, Ann Lancaster, Gordana C. Popovic, Rodrigo R. N. Rizzo, Claudia Y. Rizzo, Maria Kyriagis, Robert D. Herbert, Bart Bolsterlee

**Affiliations:** ^1^ Neuroscience Research Australia (NeuRA) Sydney New South Wales Australia; ^2^ School of Biomedical Sciences, University of New South Wales Sydney New South Wales Australia; ^3^ Cerebral Palsy Alliance Research Institute, Discipline of Child and Adolescent Health The University of Sydney Sydney New South Wales Australia; ^4^ School of Psychology, University of New South Wales Sydney New South Wales Australia; ^5^ School of Mathematics and Statistics University of New South Wales Sydney New South Wales Australia; ^6^ Evolution & Ecology Research Centre University of New South Wales Sydney New South Wales Australia; ^7^ Faculty of Medicine and Health The University of Sydney Sydney New South Wales Australia; ^8^ Stats Central, Mark Wainwright Analytical Centre University of New South Wales Sydney New South Wales Australia; ^9^ Rehab2Kids, Sydney Children's Hospital Sydney New South Wales Australia; ^10^ Graduate School of Biomedical Engineering, University of New South Wales Sydney New South Wales Australia

**Keywords:** children, growth, lower leg muscles, magnetic resonance imaging, maturation

## Abstract

Muscle volume must increase substantially during childhood growth to generate the power required to propel the growing body. One unresolved but fundamental question about childhood muscle growth is whether muscles grow at equal rates; that is, if muscles grow in synchrony with each other. In this study, we used magnetic resonance imaging (MRI) and advances in artificial intelligence methods (deep learning) for medical image segmentation to investigate whether human lower leg muscles grow in synchrony. Muscle volumes were measured in 10 lower leg muscles in 208 typically developing children (eight infants aged less than 3 months and 200 children aged 5 to 15 years). We tested the hypothesis that human lower leg muscles grow synchronously by investigating whether the volume of individual lower leg muscles, expressed as a proportion of total lower leg muscle volume, remains constant with age. There were substantial age‐related changes in the relative volume of most muscles in both boys and girls (*p* < 0.001). This was most evident between birth and five years of age but was still evident after five years. The medial gastrocnemius and soleus muscles, the largest muscles in infancy, grew faster than other muscles in the first five years. The findings demonstrate that muscles in the human lower leg grow asynchronously. This finding may assist early detection of atypical growth and allow targeted muscle‐specific interventions to improve the quality of life, particularly for children with neuromotor conditions such as cerebral palsy.

## Graphical abstract

The findings demonstrate that muscles in the human lower leg grow asynchronously. This discovery could aid in early detection of atypical growth and enable targeted muscle‐specific interventions, benefiting children with neuromotor conditions such as cerebral palsy.

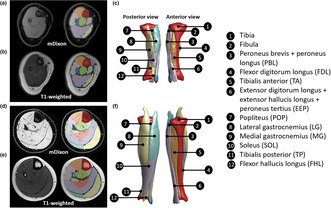

## 1 INTRODUCTION

The volume of a skeletal muscle is a crucial determinant of its functional capacity (Gans & Bock, 1965; Lieber & Fridén, 2000). Specifically, muscle volume determines the maximum power that a muscle can generate (Barrett & Harrison, 2002; O'Brien et al., 2009). Other factors such as muscle fibre composition (Lievens et al., 2020), architecture (Thom et al., 2007), and neuromuscular coordination also determine muscle power (Reid & Fielding, 2012).

Muscle volume increases substantially with growth from infancy to adulthood. A fundamental question concerns whether the growth of muscles is synchronous; that is, whether, over any period of growth, all muscles experience the same relative increase in muscle volume. Knowledge of growth rates in typically developing children may have implications for understanding disordered growth, for example, in children with neurological disorders such as cerebral palsy. Currently, it is largely unknown if growth rates differ between muscles, because most studies of muscle growth in childhood have investigated only one muscle (Bell et al., 2021; Benard et al., 2011; Binzoni et al., 2001; De Beukelaer et al., 2023; D'Souza et al., 2019; Herskind et al., 2016; Morse et al., 2008; Walhain et al., 2023; Weide et al., 2015, 2020) or a few muscles (Böl et al., 2017; Handsfield et al., 2022; Modlesky & Zhang, 2020; Mogi & Wakahara, 2022; O'Brien et al., 2010; Peeters et al., 2023; Siebert et al., 2017; Williams et al., 2022; Yanagisawa et al., 2014). O'Brien et al. (2010) reported that the four components of the quadriceps muscles undergo similar relative increases in volume from childhood to adulthood. Likewise, Yanagisawa et al. (2014) found that the four components of the rotator cuff muscles undergo similar relative increases in volume during this period. These two studies suggest that thigh and shoulder muscles grow synchronously. In contrast, rabbit leg muscles were found to have different relative growth in mass (Böl et al., 2017; Siebert et al., 2017). There is, to our knowledge, no data on the synchronicity of human lower leg muscle growth which span the period from birth to adulthood.

Most in vivo measurements of human muscle volumes have been conducted using three‐dimensional ultrasound imaging (Bell et al., 2021; Peeters et al., 2023; Walhain et al., 2020; Weide et al., 2020; Williams et al., 2021) but ultrasound imaging is more difficult with deep muscles so its application in studies of muscle growth has been largely limited to superficial muscles. Some studies have used magnetic resonance imaging (MRI; Bell et al., 2021; D'Souza et al., 2019; Handsfield et al., 2014; Handsfield et al., 2016; Handsfield et al., 2017; Morse et al., 2008; Noble et al., 2014; Noble et al., 2017; O'Brien et al., 2010; Oberhofer et al., 2010; Pitcher et al., 2018; Vanmechelen et al., 2018; Yanagisawa et al., 2014) making it possible to measure the volume of all muscles. Even so, most MRI studies have analysed only a small number of muscles because the process of manually segmenting many muscles from a single participant, or of just one or two muscles from a large cohort, is very time‐consuming (Domroes et al., 2021). The development of robust methods for automated muscle segmentation using deep learning of convolutional neural networks, a form of artificial intelligence, has made it more feasible to segment large numbers of muscles from large cohorts (Ding et al., 2020; Isensee et al., 2021; Ni et al., 2019; Zhu et al., 2021).

The aim of this study was to determine whether human lower leg muscles grow synchronously or asynchronously in typically developing children. We tested the hypothesis that human lower leg muscles grow synchronously by examining whether the volume of individual lower leg muscles, expressed as a proportion of total leg muscle volume, remains constant with age. This study is part of a larger, ongoing longitudinal investigation into childhood muscle growth (Herbert et al., 2019). This present study provides the first normative reference data set in typically developing children, which will enable the advancement of the evidence base in children with known impaired muscle growth, such as cerebral palsy.

## 2 METHODS

### 2.1 Participants

A total of 210 typically developing children (nine infants born full‐term and aged less than 3 months and 201 children aged 5 to 15 years) were recruited for the study. Infants and children aged 3 months to 5 years were excluded because it can be difficult to keep them still during an MRI scan (Spann et al., 2022). A detailed description of the muscle architecture of the eight infants has been published elsewhere (Chow et al., 2023). Muscle volume data from those infants are also reported here to enable examination of growth over the period from soon after birth to late adolescence.

Written informed consent was obtained from a parent of each participant. All procedures conformed to the Declaration of Helsinki and were approved by the Sydney Children's Hospital Network Human Research Ethics Committee (HC number: 2019/ETH11705).

### 2.2 Image acquisition

Anatomical MRI (T1‐weighted and/or mDixon) images were acquired from one randomly selected lower leg of each child. T1‐weighted scans were chosen because they are more commonly used in musculoskeletal studies and have superior contrast in identifying connective tissues such as aponeurosis and tendons (Morse et al., 2008; Pitcher et al., 2018; Williams et al., 2022). In contrast, mDixon scans were included due to their superior ability to identify bones, enhance muscles with high‐fat density, and quantify fat content (D'Souza et al., 2020; Grimm et al., 2018). The acquisition procedures for the infants (under 3 months) and older children (5–15 years) differed slightly due to the differences in the imaging strategies required for these age groups (Spann et al., 2022).

The procedures for imaging infants have been described in detail elsewhere (Chow et al., 2023) so are described only briefly here. To ensure natural sleep, each infant was fed, securely wrapped in a blanket (Antonov et al., 2017), and positioned supine in a 3T MRI scanner (Philips Ingenia CX, Philips Healthcare, Best, The Netherlands) with the legs positioned as close to extended as possible and the ankle in a relaxed position. To prevent the legs from crossing and moving, a foam wedge was placed between the legs and the legs were firmly secured with a 16‐channel small extremity coil. To protect hearing, infants were given three levels of auditory protection, including mouldable ear putty, adhesive earmuffs, and conventional earmuffs (Lundh et al., 2006).

Settings for the mDixon scans were as follows (values indicate range between participants): 2‐point 3D T1‐Fast Field Echo (T1FFE) sequence, TR = 4.2–12.5 ms, TE1 = 1.5–3.5 ms, TE2 = 2.7–4.6 ms, number of signal averages (NSA) = 1–2, acquisition voxel size = 1 × 1 × 2 mm (reconstructed to 1 × 1 × 1 mm), acquisition matrix = 160 × 160 (reconstructed to the same matrix size), number of slices = 120–330, and scan duration = 40.3–219.5 s. The settings for the T1‐weighted scans were as follows: 2D turbo spin‐echo (TSE) sequence, TR = 598.0–732.6 ms, TE = 14.7–15.5 ms, NSA = 2–3, acquisition voxel size = 0.7 × 0.83 × 5 mm (reconstructed to 0.4 × 0.4 × 5 mm), acquisition matrix = 228 × 192 (reconstructed to 400 × 400), number of slices = 24–50, and scan duration = 51.3–205.3 s.

Older children (aged 5 to 15) were placed supine in the same MRI scanner and given two levels of auditory protection. A soft foam wedge was placed under the knee of the child's selected leg to prevent compression of the calf muscles by the MRI table and hold the knee in 30° flexion. A custom‐built adjustable footplate was used to support the ankle in 15° plantarflexion. A 32‐channel torso coil was securely fastened over the child's lower legs. The child was asked to stay still throughout the scan.

The scan settings for the mDixon scans of children were the same as those planned for the infants except (values indicate range between participants): TR = 6.0–6.2 ms, TE1 = 3.5 ms, TE2 = 4.6 ms, NSA = 2, number of slices = 290–485, and scan duration = 164.1–275.7 s. The settings for the T1‐weighted scans were the same as for infants except: TR = 629.1–737.6 ms, TE = 12 ms, NSA = 2, acquisition voxel size = 0.7 × 0.85 × 5 mm (reconstructed to 0.4 × 0.4 × 5 mm), number of slices = 58–97, and scan duration = 132.8–245.2 s.

Muscles are nearly isovolumetric during contraction and passive joint rotation (Baskin & Paolini, 1967). Therefore, variations in joint position between infants and older children do not contribute to variation in muscle volume.

### 2.3 Image registration

Small misalignments between the mDixon and T1 scans were corrected using a FLIRT registration library (Greve & Fischl, 2009) implemented in MRtrix version 3.0.4 (Tournier et al., 2019). The T1 image was aligned with the corresponding mDixon image using a rigid transformation. The alignment was visually inspected by overlaying the scans with ITK‐SNAP (Yushkevich et al., 2006). Images with obvious movement artefacts were excluded from further analysis.

### 2.4 Muscle volume and bone length

**FIGURE 1:**
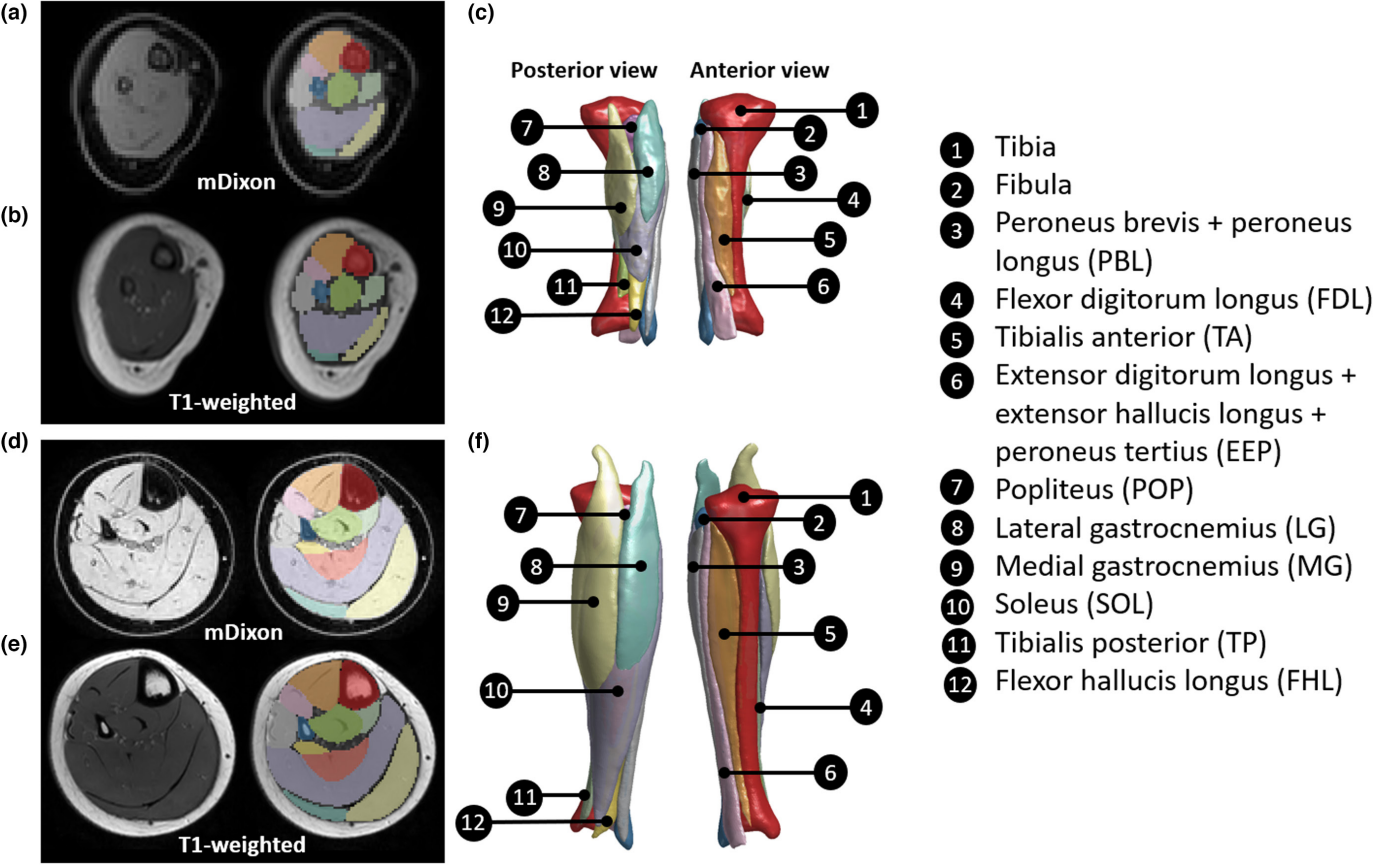
Example scans, segmentation labels and 3D surface reconstructions of lower leg muscles and bones of (a‐c) an infant aged 2 months and (d‐f) a child aged 11 years. (a and d) Transverse slice of an mDixon water image, approximately mid‐way between the knee and ankle joint, with segmentation labels overlayed in the right panel. (b and e) Transverse slice of a T1‐weighted image, same slice levels as the mDixon images in a and d, with segmentation labels overlayed in the right panel. (c and f) Posterior and anterior views of the 3D surface models.

The mDixon scan was used to segment muscles and bones (tibia and fibula) because it provides better contrast between muscles and bones than T1‐weighted scans. In cases where a participant only had a T1‐weighted scan available (*n* = 4), that scan was used for segmentation. Some small muscles were grouped together so that in total 10 muscle labels were segmented (Figure 1). The plantaris muscle was not included because it could not be identified consistently, either because it was too small in some children or because it was absent, as in some adults (Simpson et al., 1991). To account for the differences in muscle compositions between infants and older children, muscle segmentations included all tissues that were within the muscle boundaries, including blood vessels, internal aponeuroses, and fat. Similarly, to address the variations in bone compositions between infants and older children, bone segmentations included the physes (or growth plate) and the epiphyses (the rounded ends of the bones, which consist of mostly cartilaginous tissues in younger children).

Due to the significant amount of time required for manual slice‐by‐slice segmentation (~10 hours per participant), a deep learning approach was used to automate the segmentation. Building on previous work from our group in automated segmentation (Zhu et al., 2021), an nnU‐Net convolutional neural network (Isensee et al., 2021) was trained using deep learning to automate the segmentation of muscle boundaries in both infants and older children. For the infants, the network was trained using five manually segmented scans. For the older children, the network was initially trained with 20 manually segmented scans. Segmentations of 59 scans were subsequently predicted and manually corrected using ITK‐SNAP, after which the deep learning model was retrained with 79 data sets. The remaining 121 segmentations were then predicted, visually inspected, and, where necessary, manually corrected.

The segmentation labels for muscles and bones were used to create 3D surface models in MATLAB (MATLAB R2021b, The MathWorks, Inc., Natick, Massachusetts, United States) using the iso2mesh toolbox (Tran et al., 2020). All surface models were visually inspected using MATLAB (Figure 1). Muscle volume was calculated as the volume of the muscle's surface model. Relative muscle volume was calculated by dividing muscle volume by the child's total lower leg muscle volume.

Tibia and fibula lengths were defined as the maximum distance along the bone surface model's long axis (first principal component).

### 2.5 Statistical analysis

**FIGURE 2:**
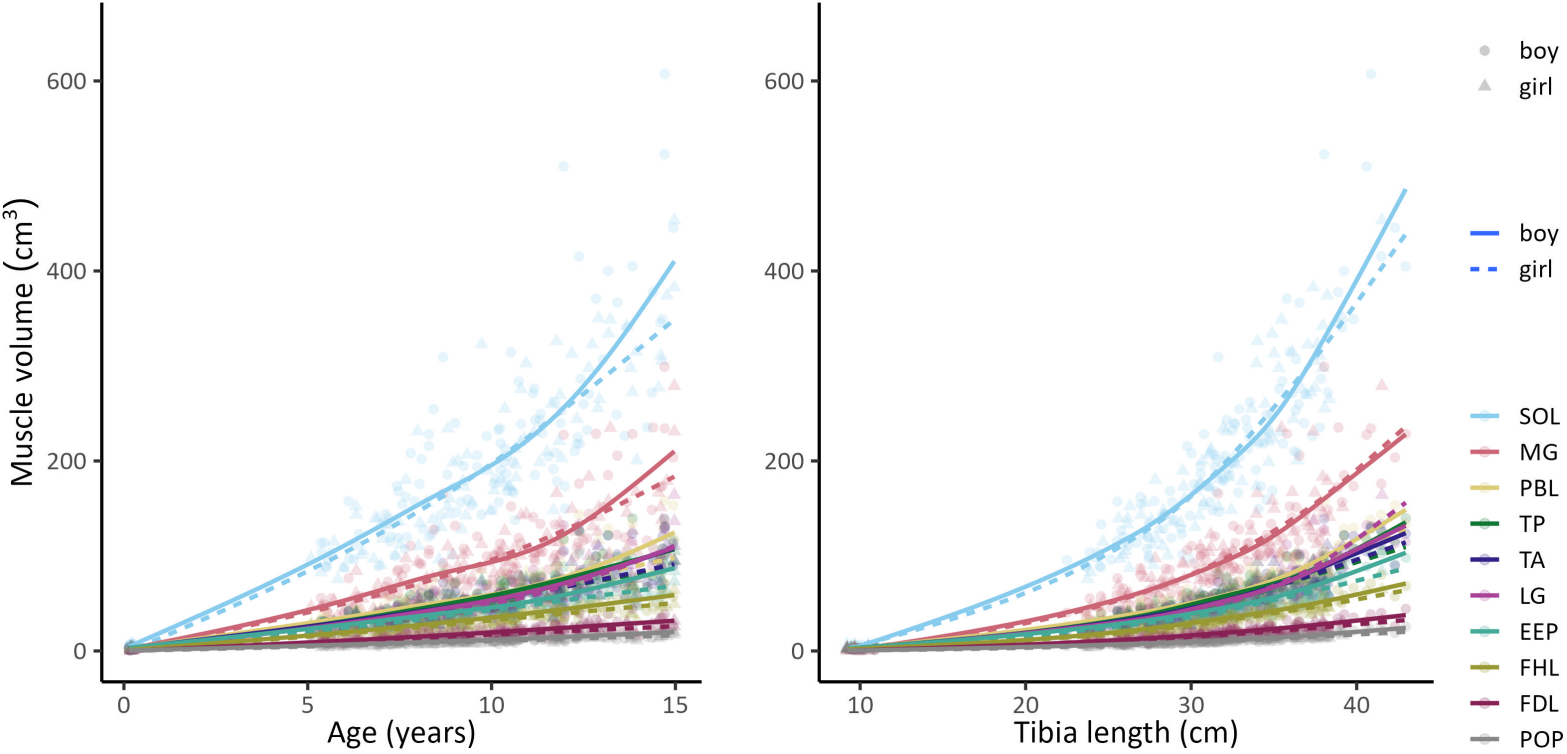
Relationship between the absolute volume of each muscle and age (left panel) and tibia length (right panel). Lines are penalised cubic regression splines. Abbreviations for muscle groups are given in Figure 1.

Statistical analyses were performed using R version 4.2.2 (R Core Team, 2022) and RStudio version 2023.6.1.524 (Posit Team, 2020). We used “ggplot2” and “mgcv” packages (Wickham, 2016; Wood, 2011) in R to fit penalised cubic regression splines to the relationships between sex‐specific absolute muscle volume and age and tibia length (Figure 2). These packages were selected for their ability to enable flexible and modelling of smooth non‐linear relationships without the need to specify specific functional forms.

**FIGURE 3:**
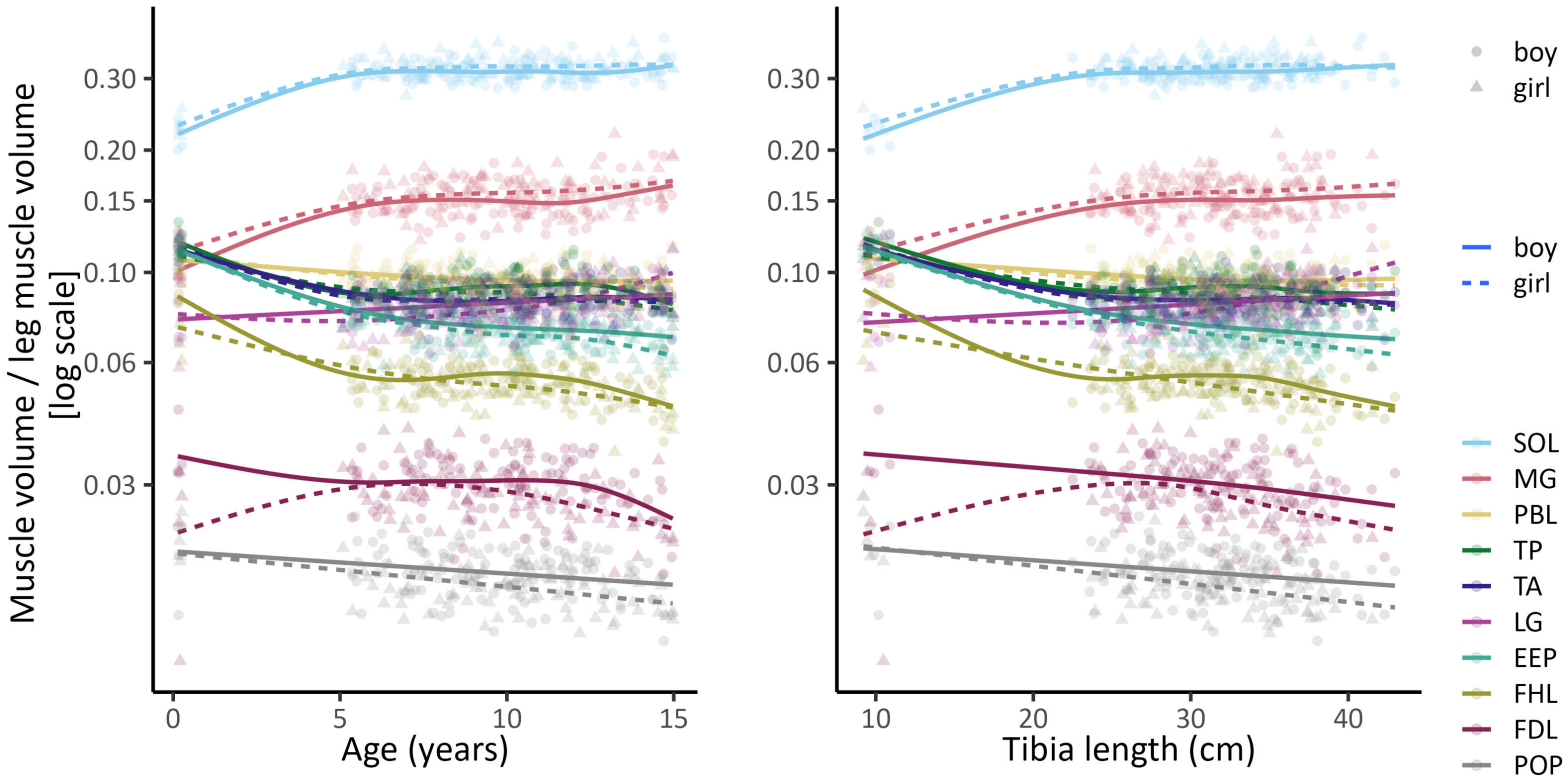
Relationship between the relative volume of each muscle and age (left panel) or tibia length (right panel). Lines are penalised cubic regression splines. Abbreviations for muscle groups are given in Figure 1.

To test whether muscles grow synchronously, we first fitted penalised cubic splines to the relationships between log‐transformed sex‐specific relative volume and age or tibia length (Figure 3) to determine whether the relative volumes remained constant with age or tibia length. Subsequently, we conducted a small‐sample test for isometry (Creasy, 1956; Jolicoeur, 1984) by fitting a major axis to each pair of log‐transformed sex‐specific absolute volumes and assessed the concordance of all the slope pairs. For this test, we used a custom‐built function written by an experienced statistician (DW).

A sensitivity analysis was conducted to determine whether there was evidence of asynchronous growth in children aged over 5 years (i.e., after exclusion of data from infants).

## 3 RESULTS

Data from two participants (aged 2.5 months and 5 years) were excluded from the analysis due to motion artefacts. So, we report data from 208 participants: eight infants under 3 months and 200 children aged 5 to 15 years. The distribution of sex did not vary appreciably with age (Table 1). Absolute muscle volumes increased nonlinearly with age and tibia length. The effect of age and tibia length on muscle volume did not differ appreciably between boys and girls (Figure 2). The relative volume of most muscles changed substantially between birth and five years, and to a lesser extent above five years (Figure 3). The test of isometry rejected the hypothesis that lower leg muscles grow synchronously (boys *p* < 0.001; girls *p* < 0.001). This indicates that muscles in the lower extremity grow asynchronously. Asynchronous growth was observed regardless of whether relative volumes were plotted against tibia length or age (Figure 3).

**TABLE 1:** Participant characteristics and lower leg volumes.

Cohort	Infants <3 months		Children [5, 7.5) years		Children [7.5, 10) years		Children [10, 12.5) years		Children [12.5, 15) years	
Sex	Boys	Girls	Boys	Girls	Boys	Girls	Boys	Girls	Boys	Girls
*n*	4	4	25	17	30	24	43	24	19	18
Age, years	0.18 ± 0.05 (0.14–0.26)	0.21 ± 0.04 (0.18–0.26)	6.50 ± 0.78 (5.36–7.46)	6.47 ± 0.69 (5.09–7.48)	8.62 ± 0.68 (7.57–9.87)	8.82 ± 0.67 (7.52–9.97)	11.13 ± 0.79 (10.02–12.42)	11.10 ± 0.67 (10.07–12.32)	13.75 ± 0.81 (12.50–14.94)	13.79 ± 0.81 (12.65–14.97)
Body mass, kg	6.7 ± 0.8 (6.0–7.9)	5.8 ± 1.3 (4.2–6.9)	23.5 ± 3.6 (17.0–32.7)	22.8 ± 3.4 (17.3–30.2)	31.1 ± 6.5 (21.3–53.5)	31.0 ± 6.6 (20.5–46.0)	39.3 ± 9.1 (24.0–76.4)	38.6 ± 6.6 (26.9–53.3)	53.1 ± 12.5 (37.0–81.9)	53.6 ± 8.1 (39.8–73.1)
Height, cm	59.1 ± 2.4 (57.0–62.5)	58.8 ± 3.6 (54.2–63.0)	120.9 ± 6.7 (108.9–133.6)	120.7 ± 6.7 (108.4–131.6)	135.2 ± 6.8 (124.8–152.0)	134.2 ± 7.3 (123.2–155.7)	147.7 ± 8.7 (130.0–172.0)	146.2 ± 7.8 (132.0–159.0)	164.4 ± 9.5 (152.0–179.7)	163.8 ± 5.0 (157.5–179.4)
Fibula length, cm	9.0 ± 0.7 (8.2–9.9)	9.0 ± 0.5 (8.6–9.7)	25.3 ± 2.0 (21.8–30.0)	25.6 ± 1.5 (22.9–28.2)	29.6 ± 2.0 (26.4–33.9)	29.2 ± 2.1 (26.7–36.2)	32.9 ± 2.4 (27.9–39.5)	32.3 ± 1.8 (29.3–35.5)	36.9 ± 2.0 (33.6–40.5)	36.1 ± 1.6 (33.9–40.8)
Tibia length, cm	10.1 ± 0.5 (9.6–10.9)	9.7 ± 0.6 (9.2–10.5)	26.1 ± 2.0 (22.5–31.0)	26.2 ± 1.5 (23.7–28.9)	30.2 ± 2.1 (26.9–34.6)	30.1 ± 2.3 (27.2–37.6)	33.9 ± 2.6 (28.9–41.5)	33.5 ± 1.9 (30.2–37.0)	38.1 ± 2.3 (34.6–43.0)	37.4 ± 1.5 (34.9–41.5)
LG volume, cm^3^	2.4 ± 0.7 (1.8–3.3)	2.2 ± 0.5 (1.7–2.9)	31.3 ± 7.0 (23.1–48.2)	27.4 ± 6.8 (17.6–39.9)	46.2 ± 13.9 (23.0–92.5)	41.2 ± 11.0 (22.0–67.3)	61.9 ± 20.1 (32.4–133.6)	58.2 ± 13.1 (33.6–81.4)	94.6 ± 20.9 (66.6–131.4)	93.4 ± 25.3 (49.7–164.3)
MG volume, cm^3^	3.2 ± 0.9 (2.3–4.3)	3.0 ± 0.6 (2.3–3.6)	56.7 ± 13.2 (40.3–85.6)	54.1 ± 13.0 (37.5–84.3)	85.3 ± 27.8 (43.6–165.7)	81.0 ± 18.5 (45.2–112.5)	105.5 ± 29.4 (70.0–227.6)	110.7 ± 25.2 (61.1–166.2)	176.8 ± 52.0 (116.2–299.2)	162.8 ± 45.4 (104.6–278.9)
SOL volume, cm^3^	6.7 ± 0.6 (6.0–7.4)	6.3 ± 1.1 (4.7–7.1)	117.9 ± 24.7 (88.1–173.8)	112.6 ± 23.9 (75.6–152.2)	173.9 ± 48.1 (101.3–309.2)	167.6 ± 49.1 (96.3–323.0)	224.7 ± 67.7 (137.7–510.0)	223.5 ± 48.4 (148.3–326.1)	346.5 ± 101.9 (226.4–607.3)	317.0 ± 56.3 (200.6–453.5)
TA volume, cm^3^	3.6 ± 0.8 (2.8–4.5)	3.2 ± 0.6 (2.4–3.7)	33.1 ± 7.1 (22.1–48.4)	30.5 ± 6.5 (21.3–39.7)	46.8 ± 10.8 (30.0–72.3)	43.6 ± 10.5 (27.8–72.5)	61.6 ± 16.6 (39.2–127.1)	61.0 ± 13.7 (35.4–93.7)	94.2 ± 24.1 (58.3–139.1)	82.4 ± 16.2 (58.3–111.2)
TP volume, cm^3^	3.7 ± 0.3 (3.3–4.0)	3.1 ± 0.4 (2.6–3.3)	33.6 ± 6.8 (24.8–52.2)	32.1 ± 6.7 (21.8–41.1)	50.7 ± 11.5 (29.8–81.5)	45.1 ± 10.2 (27.7–74.4)	66.2 ± 16.1 (41.2–125.9)	62.1 ± 10.6 (42.7–81.8)	96.2 ± 21.1 (67.0–139.7)	81.0 ± 12.7 (59.2–105.2)
EEP volume, cm^3^	3.6 ± 0.5 (3.2–4.3)	3.1 ± 0.3 (2.7–3.4)	29.5 ± 5.8 (21.0–43.0)	27.0 ± 4.6 (19.4–36.6)	41.0 ± 8.5 (26.9–60.1)	36.2 ± 7.5 (27.5–61.9)	51.8 ± 13.2 (30.4–103.1)	48.7 ± 9.3 (34.9–70.1)	76.3 ± 16.5 (54.5–103.1)	63.5 ± 10.5 (46.4–83.9)
PBL volume, cm^3^	3.4 ± 0.8 (2.6–4.5)	3.1 ± 0.7 (2.2–3.7)	37.6 ± 7.9 (27.0–55.3)	34.8 ± 6.8 (24.0–45.2)	53.1 ± 12.4 (33.3–89.5)	47.8 ± 12.6 (28.2–84.8)	68.6 ± 19.3 (44.3–143.6)	66.1 ± 12.8 (46.6–93.4)	103.7 ± 28.7 (63.2–158.2)	90.4 ± 15.5 (59.6–119.8)
POP volume, cm^3^	0.6 ± 0.2 (0.4–0.9)	0.6 ± 0.1 (0.5–0.7)	7.3 ± 1.6 (4.8–11.4)	6.2 ± 1.2 (3.9–8.1)	10.0 ± 2.3 (6.8–17.2)	8.6 ± 1.4 (6.6–12.4)	13.1 ± 3.3 (7.3–25.4)	11.7 ± 1.8 (8.6–14.8)	18.2 ± 3.5 (12.3–25.4)	15.3 ± 2.5 (11.0–20.0)
FDL volume, cm^3^	1.1 ± 0.2 (0.9–1.2)	0.6 ± 0.2 (0.3–0.8)	11.4 ± 2.2 (7.4–15.8)	11.0 ± 2.9 (6.8–17.6)	17.0 ± 3.8 (10.2–23.5)	15.0 ± 3.4 (10.6–25.1)	21.7 ± 4.6 (15.5–40.1)	19.4 ± 3.0 (14.2–25.8)	29.8 ± 6.4 (19.7–44.3)	24.3 ± 5.1 (16.0–31.8)
FHL volume, cm^3^	2.7 ± 0.5 (2.2–3.4)	2.1 ± 0.7 (1.4–3.0)	20.8 ± 4.1 (13.5–29.2)	19.7 ± 2.8 (14.6–25.0)	30.7 ± 7.1 (18.5–47.9)	26.8 ± 5.7 (19.4–44.0)	39.4 ± 9.3 (26.7–80.2)	36.9 ± 5.7 (26.6–45.8)	53.5 ± 9.2 (39.0–71.2)	46.4 ± 7.5 (32.7–64.5)
TSURAE volume, cm^3^	12.3 ± 2.0 (10.3–14.4)	11.6 ± 1.8 (9.1–13.3)	205.9 ± 43.9 (152.1–303.4)	194.1 ± 41.9 (135.1–267.5)	305.4 ± 88.1 (172.0–567.4)	289.8 ± 76.4 (163.5–502.7)	392.1 ± 115.2 (244.0–870.1)	392.4 ± 84.4 (243.0–570.7)	617.9 ± 169.0 (416.7–973.0)	573.2 ± 120.2 (354.9–896.7)
TOTAL volume, cm^3^	31.0 ± 4.9 (26.2–36.3)	27.4 ± 4.0 (21.6–30.4)	379.1 ± 75.9 (282.7–556.6)	355.3 ± 68.7 (248.3–475.3)	554.8 ± 139.1 (340.9–955.8)	512.9 ± 123.7 (327.1–877.8)	714.4 ± 189.7 (449.8–1414.2)	698.3 ± 135.7 (456.3–990.9)	1089.7 ± 263.8 (733.7–1606.7)	976.5 ± 175.1 (639.9–1432.6)

*Note*: Values are mean ± standard deviation (min–max). The square bracket “[” for the lower bound of the year range (e.g., [5, 7.5)) indicates that the endpoint is inclusive (age ≥5), while the parenthesis “)” for the upper bound indicates that the endpoint is exclusive (age <7.5). The term “TSURAE” refers to triceps surae muscle group which comprises lateral and medial gastrocnemius, and soleus muscles. The term “TOTAL” refers to all 10 muscle groups combined. All other abbreviations for muscle groups are given in Figure 1.

In the first five years, two muscles that were largest at birth (soleus and medial gastrocnemius) grew faster than other muscles, relative to initial volume (Figure 3). In contrast, the tibialis anterior, extensor digitorum longus/extensor hallucis longus/peroneus tertius (EEP) group, tibialis posterior, and flexor hallucis longus muscles grew more slowly than other muscles, relative to initial volume.

A sensitivity analysis was conducted to determine whether growth was synchronous in the older children (i.e., excluding growth in infancy). There was evidence that growth was asynchronous even when the analysis was restricted to children over five years of age (test of isometry; boys *p* < 0.001; girls *p* < 0.001).

## 4 DISCUSSION

In this study, advances in deep learning were used to determine volumes of 10 lower leg muscles from MRI scans conducted on 208 typically developing children. We found that volumes of individual leg muscles, expressed as a proportion of total lower leg muscle volume, were not constant with age, as confirmed by the test of isometry. This finding applied to both boys and girls, leading to the conclusion that human lower leg muscles grow asynchronously. Asynchronous growth appears to be most evident between 3 months and 5 years of age (Figure 3), but it continues to persist beyond 5 years of age.

Our discovery of asynchronous muscle growth in children aligns with two recent studies examining diverse age groups. One longitudinal study observed asynchronous muscle atrophy (reduction in muscle cross‐sectional area) in six muscle groups of 469 septuagenarians over a 5‐year period (Naruse et al., 2023). Another cross‐sectional study found asynchronous growth (increase in muscle volume) in four leg muscles of 118 typically developing children aged between 3 and 11 years (Peeters et al., 2023). Together, these findings suggest that age‐related changes in muscle size, whether growth in childhood or atrophy in old age, occur asynchronously across muscles. However, our findings appear inconsistent with those of O'Brien et al. (2010) and Yanagisawa et al. (2014). The former observed no significant differences in relative size among the four components of quadriceps muscles in 39 children and adults, while the latter reported no significant differences in relative size among four shoulder muscles in 40 children and adults. These discrepancies may be due to different muscle responses to other factors such as hormones, genetics, daily physical activity, and training intensity. To explore these differences, future research could consider these factors, using longitudinal designs, larger sample sizes, 3D measurements, and continuous age ranges.

Our findings more closely align with those of a series of important animal studies conducted on rabbit leg muscles (Böl et al., 2017; Siebert et al., 2017). Those studies found that the lateral gastrocnemius, medial gastrocnemius, and tibialis anterior muscles exhibited similar relative increases in mass during growth from 18 to 108 days of age, while the soleus and flexor digitorum longus muscles grew at approximately half that rate. Our study on human muscles also showed that muscles grew at different relative rates: the lateral gastrocnemius, flexor digitorum longus, and tibialis anterior muscles grew relatively more slowly than the soleus and medial gastrocnemius muscles. Of course, animal muscles are imperfect surrogates for human muscles (Binder‐Markey et al., 2023). Between‐species differences in relative growth rates of a given muscle may reflect differences in how muscles are used. For example, the relatively high growth rates of the human soleus and medial gastrocnemius muscles may reflect the outsized role of these muscles in bipedal locomotion. Understanding that muscles grow asynchronously may also have implications for understanding pathological muscle development. For instance, Handsfield et al. found that in children with cerebral palsy, while all lower limb muscles were smaller than in typically developing children, the soleus was particularly small (Handsfield et al., 2016).

The inclusion of data from 8 infants has provided valuable insights into the synchronicity of muscle growth. Our data clearly demonstrate that while asynchronous growth remains evident after 5 years when the analysis excludes the infant data, the most substantial differences in relative volumes occur in children under five years of age. Specifically, in children under five years age, the large soleus and medial gastrocnemius muscles tend to increase faster, in relative terms, than the smaller tibialis anterior, EEP group, tibialis posterior and flexor digitorum longus muscles (Figure 3). This finding was very clear, despite the relatively small number of infants in the study. We speculate that the rapid growth of the large plantarflexor muscles could be related to the onset of weight bearing and ambulation. However, for logistical reasons described above, we were unable to scan children between the ages of three months and five years, so the relative growth of muscles at the time of onset of weight bearing and ambulation is unknown. As a result, the findings during this age range should be interpreted with caution and further research is necessary to fill this data gap and confirm these findings. Alternative measurement methods, such as 3D ultrasound imaging (De Beukelaer et al., 2023; Herskind et al., 2016), may need to be used.

MRI is widely regarded as the gold standard for non‐invasive muscle volume evaluation due to its high‐resolution 3D imaging capabilities (Pons et al., 2018). However, the accuracy of individual muscle volume measurements is determined by the accuracy with which muscle boundaries can be defined. We are confident that muscle boundaries have been accurately segmented in this study. First, the segmentations were predicted by a deep learning model (Isensee et al., 2021; Zhu et al., 2021) that was trained on MRI data sets that were carefully segmented using high‐resolution data from the Visible Human data set as a reference (Ackerman, 1998). All predicted segmentations were visually verified by a human expert. Although not all muscles' volumes of children aged 5 to 15 years in this study could be compared with measurements from other studies, four muscles (medial and lateral gastrocnemius, soleus, and tibialis anterior) with similar age‐specific distributions of volumes were reported in five other MRI‐based studies (D'Souza et al., 2019; Morse et al., 2008; Oberhofer et al., 2010; Pitcher et al., 2018; Vanmechelen et al., 2018). Table S1 in the Supplementary Material provides a detailed comparison between studies. There is considerable inconsistency across studies. While some of this inconsistency may be attributable to different age distributions across studies, it seems unlikely that all of these differences can be explained by age (Obst et al., 2022). This suggests caution should be used when comparing studies that use different populations and data acquisition and analysis procedures.

Our findings were inferred from cross‐sectional data, but cross‐sectional studies of growth have important limitations. Cross‐sectional analyses of growth assume that the younger children will follow similar growth trajectories to the growth trajectories that were previously followed by the older children. That may be a reasonable assumption in the current context. A more significant limitation is that, while these cross‐sectional data show the relationship between expected muscle volume and age (or tibia length), that relationship might differ from the expected growth trajectory or the actual growth trajectory of an individual child (Obst et al., 2022). To better explore muscle growth in children, future research should utilise longitudinal designs, involving repeated measurements made on individual children over time, while also considering additional factors beyond sex, age, height, and body mass. In our ongoing project, we are currently collecting longitudinal data from this cohort (Herbert et al., 2019), but the full data set will not be available for a few more years.

The deep learning model occasionally struggled to accurately segment muscle boundaries in younger children, possibly because the thinner inter‐muscular aponeuroses in young children made the muscle identification more challenging. Segmentation errors were manually identified and corrected, prolonging processing time. Nonetheless, the deep learning model reduced the average time required to segment images from a single child substantially, from over 10 hours (full manual segmentation) to under 10 minutes per subject (prediction and manual correction). If manual segmentation were to be done without deep learning, it would have required >400 working days to complete the segmentation of 10 muscles in all 208 participants. To further improve segmentation accuracy and reduce the time required by humans to process the data, future studies could consider training the model on all currently available segmented muscles from this study, or for new studies, conducting scans with smaller voxels than were used in this study. Higher resolution scans would come at the cost of longer scan durations.

In conclusion, this study used MRI and deep learning to measure, for the first time, the volume of 10 muscle groups in a large sample of typically developing children. The data indicate that human lower leg muscles grow asynchronously, implying that growth rates are muscle specific. These data may assist early detection of atypical growth and allow targeted muscle‐specific training or rehabilitation interventions, offering improved quality of life, especially for children with neuromotor conditions such as cerebral palsy.

## AUTHOR CONTRIBUTIONS

BCVY, CM, CR, DIW, IN, GCP, MK, RDH, and BB contributed to the study's conceptualisation and methodology. BCVY, SD, AL, RRNR, CYR, RDH, and BB contributed to participant recruitment, and data collection and curation. BCVY, CR, DIW, GCP, RDH, and BB contributed to software development. BCVY, DIW, GCP, RDH, and BB contributed to data analysis and interpretation. BCVY, RDH, and BB contributed to drafting of the original manuscript. All authors contributed to the revision and approval of the final manuscript.

## CONFLICT OF INTEREST STATEMENT

I hereby state that neither I, nor any of the other authors, have had any financial or personal relationships with other people or organisations that could inappropriately influence (bias) our work.

### OPEN RESEARCH BADGES

This article has earned Open Data, Open Materials and Preregistered Research Design badges. Data, materials and the preregistered design and analysis plan are available at https://doi.org/10.17605/OSF.IO/SCFXJ.

## Supporting information


Table S1

## Data Availability

The data that support the findings of this study are available from the corresponding author upon reasonable request.
