# Supplementary material for: Human lower leg muscles grow asynchronously
Source: J Anat. 2023 Nov 2;244(3):476–85. doi: 10.1111/joa.13967 (PMC10862152; doi:10.1111/joa.13967)
Supplement: Supplementary file 1 — Table S1 [file JOA-244-476-s001.docx]

## Supplementary material

Table S1: Comparison of muscle volumes reported in the present study and in previous MRI-based studies. Values are mean ± SD (min – max). Abbreviations for muscle groups are given in Figure 1.

| Study | n (boys:girls) | Age  (years) | LG volume (cm^3^) | MG volume (cm^3^) | SOL volume (cm^3^) | TA volume (cm^3^) |
| --- | --- | --- | --- | --- | --- | --- |
| Current study | 200  (117:83) | 10.0 ± 2.6  (5.1-15.0) | 55.8 ± 26.8  (17.6-164.3) | 101.6 ± 47.9  (37.5-299.2) | 207.1 ± 91.4  (76.6-607.4) | 55.9 ± 23.6  (21.3-139.1) |
| D’Souza et al. 2019 | 20  (13:7) | 11.2 ± 3.6  ≈ (5.0-18.4) | - | 153.9 ± 74.6  ≈ (70.0-326.8) | - | - |
| Morse et al. 2008 | 11  (11:0) | 10.9 ± 0.3  ≈ (10.3-11.5) | 64.5 ± 18.9  ≈ (26.7-102.3) | - | - | - |
| Oberhofer et al. 2010 | 5  (unknown) | 10.2 ± 1.2  ≈ (7.8-12.6) | ≈ 164.0 ± 59.5  ≈ (100.0-270.0) | | ≈ 194 ± 61.2  ≈ (120.0-300.0) | - |
| Pitcher et al. 2018 | 19  (8:11) | 8 ± 1.4  ≈ (5.2-10.8) | ≈ 22.1 ± 4.3  ≈ (16.6-29.9) | ≈ 34.4 ± 6.7  ≈ (29.9-43.2) | ≈ 101.9 ± 19.9  ≈ (96.5-116.5) | ≈ 32.4 ± 6.3  ≈ (19.8-45) |
| Vanmechelen et al. 2018 | 23  (16:7) | 16.8 ± 3.3  (10.6-23.2) | - | 237 ± 57  ≈ (110.0-400.0) | 428 ± 107  ≈ (200.0-600.0) | 135 ± 41  ≈ (70.0-220.0) |
| The symbol ≈ indicates that the values were estimated based on either the reported summary statistics or by digitising data points from the graphs in the corresponding paper. | | | | | | |
